# Eltrombopag with gemcitabine-based chemotherapy in patients with advanced solid tumors: a randomized phase I study

**DOI:** 10.1002/cam4.326

**Published:** 2014-08-28

**Authors:** Eric S Winer, Howard Safran, Boguslawa Karaszewska, Donald A Richards, Lee Hartner, Frederic Forget, Rodryg Ramlau, Kirushna Kumar, Bhabita Mayer, Brendan M Johnson, Conrad A Messam, Yasser Mostafa Kamel

**Affiliations:** 1 Rhode Island HospitalProvidence, Rhode Island; 2 Brown University Oncology GroupProvidence, Rhode Island; 3 Komed Branch Medical CenterKonin, Poland; 4 Texas Oncology-TylerTyler, Texas; 5 Pennsylvania Oncology Hematology AssociatesPhiladelphia, Pennsylvania; 6 Center Hospital of the Ardenne LibramontLibramont-Chevigny, Belgium; 7 Poznan University of Medical SciencesPoznan, Poland; 8 Meenakshi Mission Hospital and Research CentreMadurai, India; 9 GlaxoSmithKlineStockley Park, United Kingdom; 10 GlaxoSmithKlineResearch Triangle Park, North Carolina; 11 GlaxoSmithKlineCollegeville, Pennsylvania

**Keywords:** Blood platelets, cancer, eltrombopag, thrombocytopenia, thrombosis

## Abstract

Preventing chemotherapy-induced thrombocytopenia could avoid chemotherapy dose reductions and delays. The safety and maximum tolerated dose of eltrombopag, an oral thrombopoietin receptor agonist, with gemcitabine-based therapy was evaluated. Patients with advanced solid tumors and platelets ≤300 × 10^9^/L receiving gemcitabine plus cisplatin or carboplatin (Group A) or gemcitabine monotherapy (Group B) were randomized 3:1 to receive eltrombopag or placebo at a starting dose of 100 mg daily administered on days −5 to −1 and days 2–6 starting from cycle 2 of treatment. Nineteen patients (Group A, *n *=* *9; Group B, *n *=* *10) received eltrombopag 100 mg and seven (Group A, *n *=* *3; Group B, *n *=* *4) received matching placebo. Nine eltrombopag patients in Group A and eight in Group B had 38 and 54 occurrences of platelet counts ≥400 × 10^9^/L, respectively. Mean platelet nadirs across cycles 2–6 were 115 × 10^9^/L and 143 × 10^9^/L for eltrombopag-treated patients versus 53 × 10^9^/L and 103 × 10^9^/L for placebo-treated patients in Groups A and B, respectively. No dose-limiting toxicities were reported for eltrombopag; however, due to several occurrences of thrombocytosis, a decision was made not to dose-escalate eltrombopag to >100 mg daily. In Groups A and B, 14% of eltrombopag versus 50% of placebo patients required chemotherapy dose reductions and/or delays for any reason across cycles 3–6. Eltrombopag 100 mg once daily administered 5 days before and after day 1 of chemotherapy was well tolerated with an acceptable safety profile, and will be further tested in a phase II trial. Fewer patients receiving eltrombopag required chemotherapy dose delays and/or reductions compared with those receiving placebo.

## Introduction

Gemcitabine is an effective treatment for solid tumors 1–4. Chemotherapy, including gemcitabine, commonly causes myelosuppression 5,6. Chemotherapy-induced thrombocytopenia (i.e., platelet counts <100 × 10^9^/L) generally necessitates gemcitabine dose delays and/or reductions, potentially compromising curative intent 5.

Eltrombopag is an oral, nonpeptide thrombopoietin receptor agonist approved for the treatment of thrombocytopenia in patients with chronic immune thrombocytopenia, and for patients with chronic hepatitis C virus-related cirrhosis to allow the initiation and maintenance of interferon-based therapy 7, and increases platelet production in patients with aplastic anemia 8 and solid tumors 9,10. Eltrombopag is also being investigated in other diseases 11–13. In preclinical studies, eltrombopag did not stimulate growth of breast, lung, or ovarian tumor cell lines at doses likely to activate megakaryocytes and megakaryocyte precursors 14. Although eltrombopag use in patients with solid tumors has been reported 9,10, eltrombopag has not been evaluated in combination with gemcitabine or gemcitabine and platinum regimens. We conducted a randomized, placebo-controlled phase I study to assess the safety and tolerability of eltrombopag, utilizing a novel dosing schedule, to determine an optimal eltrombopag dose in patients with solid tumors receiving gemcitabine as monotherapy or combined with cisplatin or carboplatin.

## Materials and Methods

### Objectives

The primary outcome was the safety and tolerability of eltrombopag given with gemcitabine-based chemotherapy. Secondary outcomes included platelet pharmacodynamics (PD), chemotherapy dose intensity and dose delays, and pharmacokinetic (PK) assessments and its relationship with plasma concentrations and PD.

### Study design

The study was conducted at centers in the United States, Europe, and India. The study protocol, any amendments, informed consent, and other information that required preapproval were reviewed and approved by a national, regional, or investigational center ethics committee or institutional review board at the participating centers. This study was conducted in accordance with the International Conference on Harmonisation Guidelines for Good Clinical Practice and all applicable patient privacy requirements, and the ethical principles that are outlined in the Declaration of Helsinki. This study is registered at www.clinicaltrials.gov (ClinicalTrials.gov identifier: NCT01147809). The protocol is available at http://www.gsk-clinicalstudyregister.com/compounds/eltrombopag#ps.

**Figure 1:**
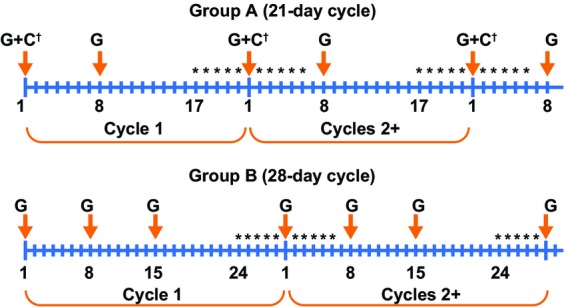
Eltrombopag and chemotherapy dosing schedules. Asterisks indicate days of eltrombopag or placebo administration. C, cisplatin or carboplatin; G, gemcitabine. ^†^Cisplatin could be divided between days 1 and 8.

All patients provided written informed consent prior to study entry. Patients were enrolled into one of two chemotherapy groups depending on whether they were receiving combination gemcitabine and platinum (cisplatin or carboplatin; Group A) or single-agent gemcitabine (Group B). To assess the safety and efficacy of eltrombopag on chemotherapy-induced thrombocytopenia when patients receive multiple cycles of gemcitabine-based chemotherapy, patients were randomized 3:1 to receive eltrombopag or matching placebo. Both investigators and patients were blinded to treatment. GlaxoSmithKline (GSK) was blinded during the conduct of the study, but not during a data review with an independent physician. Randomization, which was conducted centrally by the Registration and Medication Ordering System after each patient was registered in the study, was based on randomization schedules developed using an in-house system (RANDALL). Four dose cohorts of eltrombopag or placebo were planned (100, 150, 225, or 300 mg), with doses administered on days –5 to –1 and days 2–6 of each cycle, beginning with cycle 2 (Fig.1). Intrapatient dose escalation was not permitted. Feasibility of dose escalation was determined based on safety, tolerability, and PK data review by the sponsor and an independent, external physician, who did not participate in the study, after all patients within a cohort had completed at least two chemotherapy cycles (one cycle without and one cycle with eltrombopag/placebo) and when all patients finished all treatment cycles.

A maximum of six chemotherapy cycles were allowed: cycle 1 without eltrombopag/placebo and up to five cycles with eltrombopag/placebo. Hematology assessments (complete blood count with platelet and differential white blood cell counts) were conducted five times/cycle for Group A and six times/cycle for Group B, at study completion or early withdrawal and at the 30-day follow-up visit. Chemotherapy doses were reduced or delayed in patients with platelet counts <100 × 10^9^/L (according to the gemcitabine label). Treatment with investigational product was interrupted for patients with platelet counts ≥400 × 10^9^/L. The protocol required that thrombocytosis be considered a treatment effect rather than a dose-limiting toxicity (DLT).

A DLT was defined as a Common Terminology Criteria for Adverse Events (CTCAE) version 4.0 nonhematologic toxicity Grade ≥3 determined by the investigator to have reasonable possibility of being caused by eltrombopag. Neutropenia Grade 4 lasting for >7 days determined by the investigator to have reasonable possibility of being caused by eltrombopag may also be considered a DLT. Thrombocytopenia was considered a treatment failure rather than a DLT. Toxicities known to be caused by chemotherapy were considered DLTs if their incidence or severity was greater than expected for these regimens. Dose escalation to the next eltrombopag dose level could proceed provided a maximum of one of the six patients at a dose level receiving eltrombopag experienced a DLT. When excessive thrombocytosis occurred at the first dose level in eltrombopag-treated patients, this level was expanded to gather additional toxicity information.

Physical examinations were performed at screening, each chemotherapy dose day, and study completion or early withdrawal. Adverse events (AEs)/toxicity, including thromboembolic events (TEEs), were assessed throughout the study according to CTCAE criteria, and potential risk factors for developing TEEs were collected at study entry for all patients. Concomitant medications were monitored at screening, during the study, and up to the 6-month follow-up visit.

### Eligibility

Eligible patients were those with confirmed solid tumors scheduled to receive ≥2 cycles of gemcitabine monotherapy (1000–1250 mg/m^2^ on days 1, 8, and 15 of a 28-day cycle) or gemcitabine and platinum (gemcitabine 1000–1250 mg/m^2^ on days 1 and 8 and cisplatin 50–80 mg/m^2^ on day 1 or divided on day 1 and 8, or carboplatin area under the curve 4–7 on day 1 of each 21-day cycle). Maximum platelet counts allowed for patients during screening prior to initiation of the first planned cycle of chemotherapy were 300 × 10^9^/L. Other eligibility criteria included age ≥18 years, life expectancy ≥3 months, an Eastern Cooperative Oncology Group performance status ≤2, and adequate baseline organ function. Patients were excluded if they had preexisting cardiovascular disease; known factor V Leiden, antiphospholipid antibody syndrome, prothrombin gene mutations, low antithrombin III levels, protein C or protein S deficiency, or recent history (within 6 months) of arterial or venous thrombosis; a history of radiotherapy to more than 20% of bone marrow-bearing sites; a history of platelet agglutination abnormality, platelet disorders or dysfunction, or bleeding disorders that prevented reliable measurement of platelet counts; central nervous system metastases treated by neurosurgical resection or brain biopsy performed within 3 months; or known hepatitis B, hepatitis C, or human immunodeficiency virus. Patients with Gilbert syndrome were permitted in the study.

### Pharmacokinetic assessments

Samples for PK analysis were collected during cycle 2; one sample was taken on day 1 before the start of chemotherapy and two samples on day 4 (one sample before dosing with eltrombopag/placebo and one sample between 2 and 6 h after dosing [if administered on day 4]). Plasma eltrombopag concentration was determined using a validated analytical method 15. No formal PK or PK/PD analyses were planned at the completion of phase I of this study.

### Safety review panel

The safety review panel consisted of an independent physician not participating in the study, in addition to an internal GSK personnel group (including the GSK safety physician and the Medical Monitor). The first meeting for each group (A or B) occurred after the last enrolled patient completed study cycle 2. The second meeting occurred after the last enrolled patient completed all study treatment cycles. Both investigators and patients were blinded. While blinded during the conduct of the study, GSK was unblinded at the time of data review with the independent physician.

### Statistical analysis

Safety and efficacy data were summarized by descriptive statistics and reported using the safety population, which comprised patients who received ≥1 dose of eltrombopag or placebo.

## Results

### Patients

The study was conducted between June 2010 and January 2012. Thirty-three eligible patients were enrolled and randomized. Seven patients (Group A, *n *=* *2; Group B, *n *=* *5) were excluded from the safety and efficacy analysis as they withdrew from the study before receiving any dosing of eltrombopag or placebo, 19 (Group A, *n *=* *9; Group B, *n *=* *10) received eltrombopag 100 mg, and seven (Group A, *n *=* *3; Group B, *n *=* *4) received matching placebo. Patient demographics and disease characteristics are shown in Table1. In the eltrombopag group, eight patients (Group A, *n *=* *4; Group B, *n *=* *4) received no prior chemotherapy and 11 received prior chemotherapy, with an average of three prior regimens. All seven patients in the placebo group received prior chemotherapy, with an average of two prior regimens.

**Table 1:** Baseline demographics and disease characteristics^1^.

	Group A (gemcitabine + cisplatin/carboplatin)		Group B (gemcitabine monotherapy)	
Characteristic	Eltrombopag (*n* = 9)	Placebo (*n* = 3)	Eltrombopag (*n* = 10)	Placebo (*n* = 4)
Median age, years (range)	53 (34–75)	55 (49–56)	69 (50–74)	67.5 (31–81)
Female, *n* (%)	7 (78)	1 (33)	3 (30)	3 (75)
Platelet counts prior to starting eltrombopag or placebo (×10^9^/L), mean (SD)	108.6 (121.8)	140.0 (186.4)	269.2 (184.2)	263.7 (167.0)
Primary tumor type, *n* (%)				
Bile duct cancer/cholangiocarcinoma	3 (33)	0 (0)	0 (0)	0 (0)
Non–small cell lung cancer	1 (11)	3 (100)	4 (40)	0 (0)
Breast cancer	1 (11)	0 (0)	2 (20)	1 (25)
Colorectal cancer	1 (11)	0 (0)	2 (20)	0 (0)
Gall bladder cancer	1 (11)	0 (0)	0 (0)	0 (0)
Bladder cancer	1 (11)	0 (0)	0 (0)	0 (0)
Stomach cancer	1 (11)	0 (0)	0 (0)	0 (0)
Pancreatic cancer	0 (0)	0 (0)	2 (20)	2 (50)
Ovarian cancer	0 (0)	0 (0)	0 (0)	1 (25)
Median time since initial diagnosis, days (range)	300 (5–3621)	518.5 (280–757)	569 (13–2684)	135.5 (15–995)
Prior chemotherapy, *n* (%)				
Any	5 (56)	3 (100)	6 (60)	4 (100)
1–2	3 (33)	3 (100)	2 (20)	3 (75)
≥3	2 (22)	0 (0)	4 (40)	1 (25)

SD, standard deviation.

1 Safety population.

### Dose escalation/safety review

Eltrombopag 100 mg once daily increased platelet counts in the active treatment arms, with 92 occurrences of platelet counts ≥400 × 10^9^/L occurring in 17/19 (89%) patients in Groups A and B combined. Although thrombocytosis occurred at different points in the cycle, it mainly occurred at day 1 or just after, and tended to decrease later on within the cycle. Although no safety concerns were identified, the decision was made not to dose-escalate because of concern for extreme thrombocytosis.

### Safety

No DLTs related to eltrombopag 100 mg once daily were observed in the 19 patients receiving eltrombopag, and no safety concerns were identified by the safety review panel. AEs occurring on-therapy and for up to 30 days during follow-up are presented in Table2. Four patients receiving eltrombopag experienced liver AEs. Of the two patients in Group A, one experienced blood bilirubin increase, hypoalbuminemia, blood alkaline phosphatase increase, and alanine aminotransferase (ALT) increase (all Grade 1 or 2), and the other had an elevated liver function test (Grade 1). One patient in Group B experienced blood alkaline phosphatase increase (Grade 2), and the other patient had aspartate aminotransferase increase (Grade 1), ALT increase (Grade 1 and Grade 3), blood bilirubin increase (Grade 1), and blood alkaline phosphatase increase (Grade 2). None of these were considered by the treating physician as related to the study drug. The most common AEs in both groups were neutropenia, anemia, and thrombocytopenia.

**Table 2:** Adverse events in ≥2 patients in Group A or Group B^1^.

	Group A (gemcitabine + cisplatin/carboplatin)		Group B (gemcitabine monotherapy)	
AEs, *n* (%)	Eltrombopag (*n* = 9)	Placebo (*n* = 3)	Eltrombopag (*n* = 10)	Placebo (*n* = 4)
Any AEs	9 (100)	3 (100)	10 (100)	3 (75)
Treatment-related AEs^2^	3 (33)	2 (67)	6 (60)	1 (25)
≥Grade 3 AEs	7 (78)	2 (67)	3 (30)	2 (50)
Serious AEs	5 (56)	1 (33)	2 (20)	1 (25)
Hematologic AEs				
Leukopenia	2 (22)	1 (33)	3 (30)	2 (50)
Neutropenia	4 (44)	3 (100)	5 (50)	2 (50)
Anemia	4 (44)	1 (33)	4 (40)	1 (25)
Thrombocytopenia	3 (33)	2 (67)	3 (30)	3 (75)
Thrombocytosis	2 (22)	2 (67)	2 (20)	1 (25)
Platelet counts increased	0 (0)	0 (0)	3 (30)	0 (0)
Nonhematologic AEs				
Nausea	5 (56)	1 (33)	0 (0)	0 (0)
Vomiting	2 (22)	1 (33)	0 (0)	0 (0)
Anxiety	2 (22)	0 (0)	0 (0)	0 (0)
UTI	2 (22)	0 (0)	0 (0)	0 (0)
Fatigue	1 (11)	1 (33)	2 (20)	1 (25)
Decreased appetite	1 (11)	0 (0)	2 (20)	0 (0)
Increased alkaline phosphatase	1 (11)	0 (0)	2 (20)	0 (0)
Peripheral edema	1 (11)	0 (0)	2 (20)	0 (0)
Headache	1 (11)	0 (0)	1 (10)	1 (25)
Dyspnea	1 (11)	1 (33)	1 (10)	0 (0)
Pyrexia	1 (11)	1 (33)	0 (0)	1 (25)
Alopecia	0 (0)	0 (0)	1 (10)	1 (25)
Dizziness	0 (0)	0 (0)	1 (10)	1 (25)
AEs of special interest				
Liver AEs	2 (22)	0 (0)	2 (20)	0 (0)
Renal AEs	3 (33)	0 (0)	0 (0)	2 (50)
DVT/venous thrombosis^3^	2 (22)	0 (0)	1 (10)	0 (0)

AE, adverse event; DVT, deep vein thrombosis; UTI, urinary tract infection.

1 Safety population; on-therapy + 30 days. All toxicities were reported based on the Common Terminology Criteria for Adverse Events version 4.0.

2 Treatment-related AEs in Group A included thrombocytosis, vomiting, and thrombocytopenia in the placebo group and nausea, lymphopenia, cystitis, and thrombocytosis in the eltrombopag group. In Group B, treatment-related AEs included thrombocytosis in the placebo group, and increased platelet count, constipation, thrombocytosis, and hypercalcemia in the eltrombopag group.

3 None of these events were considered related to eltrombopag, and all resolved. One event occurred after stopping eltrombopag and following disease progression.

In both chemotherapy groups, a higher percentage of patients treated with placebo versus eltrombopag reported AEs of thrombocytopenia and neutropenia (Table2). More patients receiving eltrombopag reported anemia as an AE versus those receiving placebo. However, looking at laboratory values, a higher percentage of patients in both chemotherapy Groups A and B had Grade 3 or 4 anemia while receiving placebo versus eltrombopag (Table3). A higher percentage of patients receiving placebo also reported Grade 3 or 4 thrombocytopenia and local laboratory-reported neutropenia.

**Table 3:** Patients with Grade 3 or 4 thrombocytopenia, neutropenia, and/or anemia, based on laboratory results.

	Group A (gemcitabine + cisplatin/carboplatin)		Group B (gemcitabine monotherapy)	
	Eltrombopag (*n* = 9)	Placebo (*n* = 3)	Eltrombopag (*n* = 10)	Placebo (*n* = 4)
Thrombocytopenia, *n* (%)^1^,^2^	4 (44)	3 (100)	2 (20)	2 (50)
Thrombocytopenia, *n* (%)^1^,^3^	3 (33)	2 (67)	0 (0)	1 (25)
Neutropenia, *n* (%)^2^,^4^	4 (44)	2 (67)	0 (0)	0 (0)
Anemia, *n* (%)^1^,^2^	2 (22)	1 (33)	3 (30)	2 (50)

1 Central laboratory results.

2 Cycle 1 to the end of the 30-day follow-up.

3 Cycle 2 to the end of the 30-day follow-up.

4 Local laboratory results.

Three TEEs were reported in three patients receiving eltrombopag: two serious AEs of deep vein thrombosis in Group A (one in a patient with metastatic gall bladder cancer that occurred after computed tomography confirmation of gastric outlet obstruction and disease progression, and one in a patient with metastatic urinary bladder cancer), and one AE of venous thrombosis in Group B (in a patient with metastatic colorectal cancer, diagnosed clinically with no confirmatory laboratory or Doppler assessments). All three patients had several underlying risk factors for developing TEEs at study enrollment, including hypertension, hyperlipidemia, previous long-term history of smoking, hypercholesterolemia, history of diabetes mellitus, presence of multiple metastatic disease, cardiac problems, and dehydration. No TEEs were considered related to eltrombopag therapy by the treating physician; none required study withdrawal and all resolved.

In Group A, one death (33%) occurred in the placebo arm 63 days following the last dose, and five deaths (56%) occurred in the eltrombopag arm more than 30 days following the last dose (range, 36–231 days). In Group B, two deaths (50%) occurred in the placebo arm (one death at 18 days and one death at 40 days after the last dose) and six deaths (60%) occurred in the eltrombopag arm more than 30 days after therapy (range, 76–112 days). Three additional patients in Group B died before receiving any dose of eltrombopag or placebo. All three deaths were attributed to disease progression or disease under study.

### Platelet response

**Figure 2:**
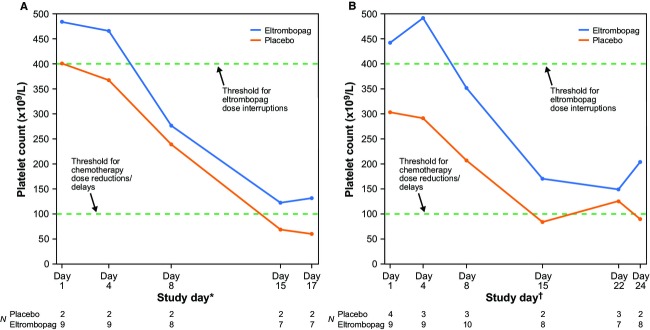
Mean platelet counts across cycles 2–6 at each visit for (A) Group A and (B) Group B. *For days 1 and 8 of chemotherapy dosing, platelet counts before chemotherapy are shown. ^†^For days 1, 8, and 15 of chemotherapy dosing, platelet counts before chemotherapy are shown.

Mean platelet counts across cycles 2 through 6 were consistently higher at each assessment visit in patients receiving eltrombopag versus placebo (Fig.2A and B). Mean platelet nadirs (standard deviation) across cycles 2–6 in Group A were 115 × 10^9^/L (83 × 10^9^/L) for eltrombopag and 53 × 10^9^/L (7 × 10^9^/L) for placebo. In Group B, these were 143 × 10^9^/L (82 × 10^9^/L) for eltrombopag and 103 × 10^9^/L (64 × 10^9^/L) for placebo.

In the eltrombopag arms, nine patients in Group A had 38 occurrences of platelet counts ≥400 × 10^9^/L and eight patients in Group B had 54 occurrences of platelet counts ≥400 × 10^9^/L; therefore, the dose of eltrombopag was not escalated beyond 100 mg once daily. In placebo-treated patients, nine occurrences of platelet counts ≥400 × 10^9^/L were reported in three patients in Group A and nine occurrences in one patient in Group B. The highest platelet counts seen were 825 × 10^9^/L for eltrombopag and 562 × 10^9^/L for placebo in Group A, and 902 × 10^9^/L for eltrombopag and 609 × 10^9^/L for placebo in Group B. Because thrombocytosis was considered a treatment effect per the protocol, many of these elevated platelet counts were not reported as AEs/serious AEs. No sequelae related to these thrombocytosis events were reported.

**Figure 3:**
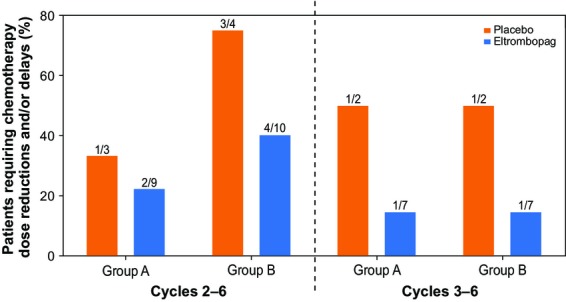
Proportions of patients requiring chemotherapy dose reductions and/or delays. The need for dose adjustments with continued chemotherapy remained lower in eltrombopag-treated patients compared with placebo-treated patients between cycles 2–6 and 3–6.

In Groups A and B, the number of patients requiring chemotherapy dose reductions and/or delays for any reason in cycles 2–6 and cycles 3–6 was lower with eltrombopag than with placebo. In patients receiving eltrombopag, only 22% in Group A and 40% in Group B experienced a reduction/delay in their chemotherapy across cycles 2–6. The corresponding figures for patients receiving placebo were 33% and 75% for Groups A and B, respectively (Fig.3). Across cycles 3–6 in each chemotherapy group (Groups A and B), 14% of eltrombopag-treated patients and 50% of placebo-treated patients required chemotherapy dose reductions/delays for any reason. The reasons for dose reductions and/or dose delays included, but were not limited to, neutropenia, thrombocytopenia, and other AEs.

### Pharmacokinetics

**Figure 4:**
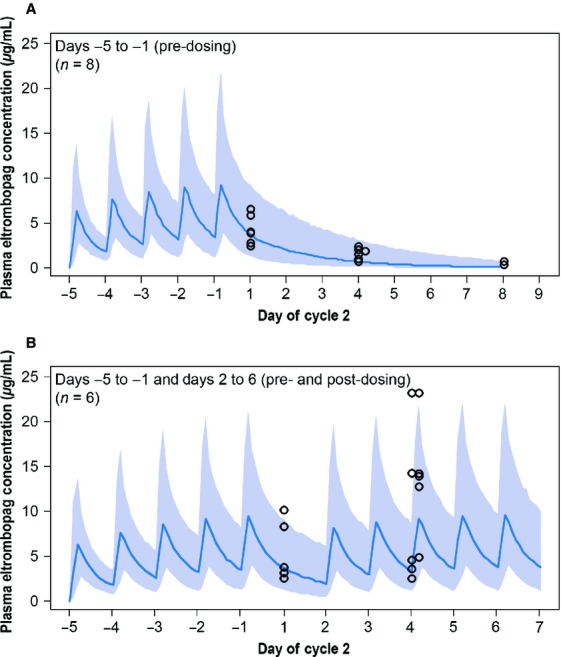
Cycle 2 plasma eltrombopag concentrations at planned time points for patients in Groups A and B receiving eltrombopag. (A) Patients who received protocol-specified predosing but not protocol-specified postdosing due to day 1 platelet counts ≥400 × 10^9^/L and (B) patients who received protocol-specified predosing and postdosing. Observed data (symbols) and 90% prediction interval (shaded, with median line) based on a previous pharmacokinetics model.

Plasma eltrombopag concentration data from cycle 2 are shown in Figure4. Although limited, the observed PK data were consistent with the expected plasma eltrombopag concentration based on a population PK analysis from a previous eltrombopag study in patients with solid tumors. The median (range) apparent oral clearance for patients receiving eltrombopag 100 mg can be calculated as 9.98 (1.89–23.5) mL/min from data presented in Hayes et al. 16, resulting in a terminal half-life of ∼32 h. A final, combined PK analysis will be presented upon completion of phase II of this study.

## Discussion

Bleeding from thrombocytopenia, or platelet transfusion requirement, is uncommon for patients receiving gemcitabine-containing regimens to treat solid tumors. However, persistence of platelet counts ≤100 × 10^9^/L frequently occurs and generally necessitates dose delays and/or reductions 6. Studies have demonstrated that Grades 3 and 4 thrombocytopenia occur in between 16% and 55% of patients receiving gemcitabine/platinum treatment regimens 17–21, and 3–16% of patients receiving single-agent gemcitabine 21–23. Previous attempts to use thrombopoietin-stimulating agents have been successful at maintaining dose intensity, although antibody formation limited the use of these agents 24.

The present study aimed to determine the optimal dose and schedule of eltrombopag given with gemcitabine chemotherapy for a phase II trial. Although a limited number of patients were enrolled in this phase I study, more dose reductions/delays (for any reason including thrombocytopenia) occurred in placebo-treated patients than in eltrombopag-treated patients. This potentially clinically significant result will be investigated further in the phase II trial, as chemotherapy-induced thrombocytopenia could be detrimental to outcome when gemcitabine-based chemotherapy is used for curative intent 1,2,4.

In this study, the first dose level of eltrombopag 100 mg once daily was well tolerated. Although the safety review panel identified no safety concerns, the decision was made not to dose-escalate eltrombopag due to the increased risk of thrombocytosis (number of events and absolute platelet counts). Three instances of deep venous thrombosis were noted; however, this incidence is similar to what is seen in previous gemcitabine 25 and gemcitabine/platinum-based regimens, and its occurrence in the eltrombopag arm is consistent with the 3:1 randomization of patients receiving eltrombopag versus placebo. All patients who developed a TEE had multiple baseline risk factors for its development. In addition, some of these patients were heavily pretreated or had a diagnosis of disease progression before the development of the TEE. It is not the standard of care to anticoagulate such patients as this would increase the risk of bleeding 26. None of the TEEs observed in this study were considered related to eltrombopag by the treating physicians. Studies of the action of eltrombopag on platelets indicate that eltrombopag does not activate platelets 27,28.

Kellum et al. 9 reported that administration of eltrombopag (50, 75, or 100 mg once daily) for 10 days following chemotherapy (days 2–11; carboplatin and paclitaxel on day 1 of 21-day cycles) in 183 patients with solid tumors resulted in higher mean platelet counts on day 1 (the day of chemotherapy administration) of cycles 2 and 3 compared with placebo. In that study, post-nadir platelet counts increased during cycles 1 and 2 with eltrombopag versus placebo, resulting in higher platelet counts at the start of the next chemotherapy treatment cycle 9. Although results of the Kellum et al. 9 study were promising, the primary end point of reducing the platelet count from day 1 in cycle 2 to the platelet nadir in cycle 2 was not reached, suggesting the dosing schedule of eltrombopag was not optimal.

In the current study, patients received eltrombopag for 5 days before and after day 1 of each chemotherapy cycle (−5/+5 schedule), starting in cycle 2. This schedule was based on PK/PD modeling 16 using data from Kellum et al. 9 and preclinical and clinical studies of recombinant human thrombopoietin supportive therapy 14,29. Those data suggest administration of recombinant human thrombopoietin both before and after chemotherapy maximizes efficacy 29. PK/PD simulations using a model of eltrombopag in chemotherapy-induced thrombocytopenia also suggest that administration for 10 days either before or after chemotherapy administration may exaggerate fluctuations in platelet counts, and improved platelet count profiles would be achieved using the −5/+5 eltrombopag dosing schedule 16. The efficacy of eltrombopag 100 mg in this study appears to be greater than that reported by Kellum et al. 9, which may be explained, at least in part, by the −5/+5 eltrombopag dosing schedule. It is intriguing that eltrombopag maintained average platelet counts >100 × 10^9^/L across cycles 2 through 6 at each time point, including the nadir. Interestingly, eltrombopag-treated patients had lower incidences of Grades 3 and 4 neutropenia (from local laboratory results), anemia, and thrombocytopenia. This was also seen in Kellum et al. 9.

This trilineage effect may be similar to what was reported previously with eltrombopag in patients with aplastic anemia 8. Hematopoietic stem cells and progenitor cells express the thrombopoietin receptor c-MPL. In patients with aplastic anemia, eltrombopag increased neutrophils, red blood cells, and platelets, suggesting trilineage hematopoiesis 8.

It was also clinically meaningful to realize that fewer patients receiving eltrombopag required chemotherapy dose delays and/or reductions for any reason compared with those receiving placebo. This was confirmed when analyzing cycles 2–6 as well as cycles 3–6 (Fig.3).

A limitation to this study is the relatively small number of patients enrolled, especially in the placebo arm, which limited comparisons between the eltrombopag and placebo arms. However, the safety data together with the preliminary efficacy results, especially the platelet elevation noted in the eltrombopag arm to >400 × 10^9^/L in many instances, were a promising sign of activity.

In conclusion, eltrombopag was generally well tolerated with no unexpected AEs. In both chemotherapy groups, eltrombopag 100 mg once daily administered 5 days before and after initiation of gemcitabine-based chemotherapy may have ameliorated thrombocytopenia. To our knowledge this is the first thrombopoietin receptor agonist showing positive results for patients with solid tumors receiving gemcitabine-based therapy. Based on these findings, the eltrombopag dose of 100 mg once daily (−5/+5 dosing schedule) was chosen for a subsequent phase II study of eltrombopag versus placebo in thrombocytopenic patients receiving gemcitabine-based chemotherapy, which is ongoing.
